# Low levels of Stat5a protein in breast cancer are associated with tumor progression and unfavorable clinical outcomes

**DOI:** 10.1186/bcr3328

**Published:** 2012-10-04

**Authors:** Amy R Peck, Agnieszka K Witkiewicz, Chengbao Liu, Alexander C Klimowicz, Ginger A Stringer, Edward Pequignot, Boris Freydin, Ning Yang, Adam Ertel, Thai H Tran, Melanie A Girondo, Anne L Rosenberg, Jeffrey A Hooke, Albert J Kovatich, Craig D Shriver, David L Rimm, Anthony M Magliocco, Terry Hyslop, Hallgeir Rui

**Affiliations:** 1 Department of Cancer Biology, Kimmel Cancer Center, Thomas Jefferson University, 233 S. 10th Street, Philadelphia, PA 19107, USA; 2 Department of Pathology, Thomas Jefferson University, 132 S. 10th Street, Philadelphia, PA 19107, USA; 3 Department of Oncology, University of Calgary, 1331 29th St NW, Calgary, Alberta T2N 4N2, Canada; 4 Division of Biostatistics, Thomas Jefferson University, 1015 Chestnut St, Philadelphia, PA 19107, USA; 5 Department of Surgery, Thomas Jefferson University, 1100 Walnut Street, Philadelphia, PA 19107, USA; 6 Breast Center, Walter Reed National Military Medical Center, 8901 Wisconsin Ave, Bethesda, MD 20889, USA; 7 Department of Surgery, Walter Reed National Military Medical Center, 8901 Wisconsin Ave, Bethesda, MD 20889, USA; 8 MDR Global Systems, LLC, 425 Park Place, Windber, PA 15963, USA; 9 Department of Pathology, Yale University School of Medicine, 310 Cedar St, New Haven, CT 06520, USA; 10 Department of Anatomical Pathology, H. Lee Moffitt Cancer Center and Research Institute, 12902 Magnolia Drive, Tampa, FL 33612, USA

## Abstract

**Introduction:**

Signal transducer and activator of transcripton-5a (Stat5a) and its close homologue, Stat5b, mediate key physiological effects of prolactin and growth hormone in mammary glands. In breast cancer, loss of nuclear localized and tyrosine phosphorylated Stat5a/b is associated with poor prognosis and increased risk of antiestrogen therapy failure. Here we quantify for the first time levels of Stat5a and Stat5b over breast cancer progression, and explore their potential association with clinical outcome.

**Methods:**

Stat5a and Stat5b protein levels were quantified *in situ *in breast-cancer progression material. Stat5a and Stat5b transcript levels in breast cancer were correlated with clinical outcome in 936 patients. Stat5a protein was further quantified in four archival cohorts totaling 686 patients with clinical outcome data by using multivariate models.

**Results:**

Protein levels of Stat5a but not Stat5b were reduced in primary breast cancer and lymph node metastases compared with normal epithelia. Low tumor levels of Stat5a but not Stat5b mRNA were associated with poor prognosis. Experimentally, only limited overlap between Stat5a- and Stat5b-modulated genes was found. In two cohorts of therapy-naïve, node-negative breast cancer patients, low nuclear Stat5a protein levels were an independent marker of poor prognosis. Multivariate analysis of two cohorts treated with antiestrogen monotherapy revealed that low nuclear Stat5a levels were associated with a more than fourfold risk of unfavorable outcome.

**Conclusions:**

Loss of Stat5a represents a new independent marker of poor prognosis in node-negative breast cancer and may be a predictor of response to antiestrogen therapy if validated in randomized clinical trials.

## Introduction

Signal transducer and activator of transcription-5a (Stat5a) was first identified as "mammary gland factor" [1], and subsequently, the highly homologous but distinct *Stat5b *gene was discovered [2]. Stat5a and Stat5b are activated in response to hormones or cytokines by phosphorylation of a tyrosine residue located within structurally identical motifs near their C-terminal transactivation domains. Phosphorylated Stat5 molecules undergo functional dimerization, nuclear translocation, and DNA binding to modulate expression of target genes that promote mammary epithelial cell survival, proliferation, and differentiation [3-5]. Stat5a and Stat5b are encoded by separate genes but share greater than 90% amino acid identity. Despite some overlapping functions, Stat5a and Stat5b have distinct regulatory features and functions [6-8], as well as distinct tissue-specific expression patterns [4,5]. Based on gene-knockout studies in mice, pregnancy-associated growth and differentiation of normal mammary epithelia require Stat5a but not Stat5b [4,9]. However, consistent with partially overlapping functions of Stat5a and Stat5b, Stat5b is phosphorylated during pregnancy, and upregulation of phospho-Stat5b in Stat5a-deficient mice is associated with restored lactation after repeated pregnancies [5,10]. Emerging evidence points to critical involvement of Stat5 transcription factors in the development and progression of breast cancer. Current data support the concept of dual roles of Stat5a/b proteins as promoters of mammary tumorigenesis, and as suppressors of the progression of established breast cancer [11,12], although our knowledge of individual roles of Stat5a and Stat5b in breast cancer remains rudimentary.

Experimental support for a promoting role of Stat5a in mammary tumor initiation includes genetic models in mice in which Stat5a is either suppressed or hyperactivated [13-15]. A function for Stat5a in tumor initiation may involve proliferative upregulation of cyclin D1 [16,17] and antiapoptotic effects, for instance, through upregulation of Akt1 [18] or induction of Survivin [19]. Conversely, experimental *in vitro *data indicate that Stat5a maintains cellular differentiation and suppresses epithelial-to-mesenchymal transition and invasive characteristics of human breast cancer cell lines [20-23], and a positive association between nuclear Stat5a and more well-differentiated human breast cancer has been reported [24,25]. Expression of constitutively active Stat5a promoted breast cancer cell survival and anchorage-independent growth but inhibited migration, whereas constitutively active Stat5b had little or no effect, possibly because of its limited capacity to upregulate Survivin [19]. Furthermore, prolactin-suppression of the *Bcl6 *oncogene in human breast cancer cell lines [26] was preferentially mediated by Stat5a over Stat5b [22]. In contrast, Stat5b has been reported to contribute to the progression of established breast cancer. Stat5b but not Stat5a promoted *in vitro *migration of ER-negative BT549 and MDA-MB-231 breast cancer cells [27]. In ER-positive T47D and MCF7 cells, Stat5b was implicated as a mediator of estrogen-induced proliferation, and a constitutively active Stat5b mutant induced resistance to antiestrogens [28]. Collectively, these experimental data indicate distinct roles of Stat5a and Stat5b in breast tumor biology.

In healthy human and rodent mammary epithelia outside of pregnancy, Stat5a/b is consistently tyrosine phosphorylated and transcriptionally active at a basal level [5], whereas a progressive loss of nuclear-localized, tyrosine-phosphorylated Stat5a/b (Nuc-pYStat5a/b) has been observed in invasive and metastatic breast cancer [22,29,30]. Low levels of Nuc-pYStat5a/b were a strong independent marker of poor prognosis in node-negative breast cancer and were also a strong independent marker associated with antiestrogen therapy failure [29,30]. Importantly, phosphotyrosine motifs surrounding the conserved amino acid residues Y694 of Stat5a and Y699 of Stat5b are identical and cannot be distinguished by phosphotyrosine-Stat5 antibodies. Most analyses of Stat5 in breast cancer specimens to date have focused on detecting either Stat5a or Stat5b or investigated Stat5 without discriminating between Stat5a and Stat5b. As a result, apparently conflicting lines of evidence have been reported, based on a few limited studies for associations between clinicopathologic parameters and levels of Stat5 transcription factors. Furthermore, the mechanisms underpinning loss of Nuc-pYStat5a/b in breast cancer remain to be identified, and it is unknown whether reduced Nuc-pYStat5a/b levels in breast cancer may in part reflect reduced levels of Stat5a and/or Stat5b protein expression.

Several studies have reported low levels of Stat5 protein in invasive breast cancer. First, only 34% of 517 invasive breast cancer cases were positive for epithelial cell Stat5 expression, as measured by immunohistochemistry, and nuclear Stat5 was detected in as few as 18 (3%) of cases [31]. In that study, undetectable Stat5 protein was associated with poor clinical outcome in ER-positive patients and poor response to post-relapse antiestrogen therapy [31].

Second, cytoplasmic or nuclear Stat5a was detected in 17% of 30 cases of breast adenocarcinomas compared with nearly 100% positive expression in normal tissue [25]. In contrast, our initial study of a breast cancer progression material by using a pan-Stat5a/b antibody indicated that malignant breast tumors generally remained positive for expression of Stat5 protein across the progression series, although quantification was not attempted, and the antibody used did not discern between Stat5a and Stat5b [29]. Other groups reported high frequencies of nuclear Stat5a expression in breast adenocarcinomas, ranging from 48% to 74% of cases [24,32]. Collectively, these discrepant and incomplete data warrant a more systematic effort to quantify Stat5a or Stat5b protein expression during human breast cancer progression relative to normal breast tissue.

With selective Stat5a and Stat5b antibodies and immunofluorescence detection on the Automated Quantitative Analysis (AQUA) platform [33,34], Stat5a and Stat5b protein levels were quantified in breast cancer progression material. Unexpectedly, the analyses revealed frequent and selective loss of nuclear as well as total Stat5a protein in invasive breast cancer and lymph node metastases, whereas expression of Stat5b remained unchanged. Low transcript levels for Stat5a but not Stat5b in breast cancer specimens were associated with poor clinical outcome. Based on four independent clinical materials, reduced levels of nuclear-localized Stat5a were prognostic of unfavorable breast cancer outcome in patients who did not receive systemic adjuvant therapy and were associated with elevated risk of failure of antiestrogen therapy in patients. Loss of Stat5a protein may represent a new mechanism contributing to the reported frequent loss of Nuc-pYStat5a/b during breast cancer progression.

## Materials and methods

### Paraffin-embedded breast tumor specimens

Five independent and deidentified clinical cohorts of breast cancer tissues, represented as formalin-fixed and paraffin-embedded whole tissue sections or tissue microarrays, were evaluated. The research use of tissues was approved by the ethics committee of the respective institutions, and informed consent was waived as anonymous archival tissue specimens were used. Clinical information was not available for progression Material I. Patient demographics and clinical features of Materials III, IV, V, and VI are presented in Table 1.

**Table 1:** Characteristics of patients in Materials III, IV, V, and VI

		Material III prognosis (*n *= 233)		Material IV prognosis (*n *= 291)		Material V antiestrogen (*n *= 75)		Material VI antiestrogen (*n *= 97)	
		Number	%	Number	%	Number	%	Number	%
Center	Fox Chase	64	27	-	-	19	25	-	-
	Kaiser	79	34	-	-	35	47	-	-
	Univ of Miami	30	13	-	-	11	15	-	-
	Washington Univ	60	26	-	-	10	13	-	-

Race	Asian	1	0.4	0	0	1	1.3	-	-
	Black	16	7	3	1	6	8	-	-
	White	216	93	287	99	67	89	-	-
	Other	0	0	1	0.3	1	1.3	-	-

Age (years)	< 50	42	18	86	30	9	12	6	6
	≥50	191	82	205	70	66	88	91	94

Size (cm)	< 2	117	50	114	39	41	55	39	40
	≥2 to < 5	107	46	133	46	34	45	43	44
	≥5	9	4	33	11	0	0	11	11
	Missing	0	0	11	4	0	0	4	4

Grade	1	61	26	67	23	17	23	14	14
	2	104	45	137	47	45	60	47	48
	3	68	29	48	16	13	17	32	33
	Missing	0	0	39	13	0	0	4	4

ER status	Negative	44	19	97	33	1	1.3	11	11
	Positive	186	80	163	56	72	96	80	82
	Missing	3	1.3	31	11	2	3	6	6

PR status	Negative	65	28	103	35	9	12	-	-
	Positive	122	52	151	52	56	75	-	-
	Missing	46	20	37	13	10	13	-	-

ER/PR status	Negative	44	19	68	23	1	1.3	12	12
	Positive	178	76	192	66	72	96	83	86
	Missing	11	5	31	11	2	3	2	2

HER2 status	Negative	-	-	217	75	-	-	77	79
	Positive	-	-	33	11	-	-	13	13
	Missing	-	-	41	14	-	-	7	7

Nodal status	Negative	233	100	291	100	73	100	42	43
	Positive	0	0	0	0	0	0	42	43
	Missing	0	0	0	0	0	0	13	13

Chemotherapy	Untreated	233	100	-	-	75	100	97	100
	Treated	0	0	-	-	0	0	0	0
	Missing	0	0	-	-	0	0	0	0

Hormone therapy	Untreated	233	100	-	-	0	0	0	0
	Treated	0	0	-	-	75	100	97	100

Radiation therapy	Untreated	187	80	-	-	48	64	36	37
	Treated	46	20	-	-	27	36	59	61
	Missing	0	0	-	-	0	0	2	2

Stat5a status	Low	30	13	200	69	9	12	24	25
	High	193	83	39	13	64	85	47	48
	Missing	10	4	52	18	2	3	26	27

CSS events	Number	52	22	107	37	10	13	56	58

TTR events	Number	56	24	-	-	13	17	60	62

Evaluable subjects for multivariate	Number	218	94	190	65	73	97	55	57

		**Mean (Range)**		**Mean (Range)**		**Mean (Range)**		**Mean (Range)**	

Stat5a score	-	32 (0-90)		653 (175-2,099)		34.1 (0-80)		1,949 (727-4,626)	

Date of diagnosis	Year	1974-1990		1953-1980		1986-1996		1990-2000	

Age at diagnosis	Years	62.3 (31-88)		57.3 (24-86)		64.6 (43-88)		69.8 (38-89)	

Tumor size	cm	2.1 (0.6-7.5)		2.5 (0.4-11)		1.9 (0.5-4.5)		2.7 (0.4-11)	

Follow-up	Months	126 (3-326)		160 (1-425)		117 (10-195)		41 (4-143)	

ER, estrogen receptor; PR, progesterone receptor; SD, standard deviation.

Material I was a breast cancer progression tissue array constructed by using cutting-edge matrix assembly [35] and represented 180 unmatched patient specimens, including 40 normal breast tissues, 20 ductal carcinoma *in situ *(DCIS), 100 invasive ductal carcinomas (IDCs), and 20 lymph node breast cancer metastases [22]. Tissues were obtained from Thomas Jefferson University Hospital archives. The array contained 59% estrogen receptor (ER)-positive, 42% progesterone receptor (PR)-positive, and 20% Her2-positive cases, as determined by pathologist scoring of standard DAB-chromogen immunohistochemistry (DAB-IHC). Stat5a scores, detected by immunofluorescence and quantified by automated quantitative analysis (AQUA), were obtained for 126 cases, and Stat5b AQUA scores were obtained for 116 cases.

Material III, obtained through the National Cancer Institute Cooperative Breast Cancer Tissue Resource, comprised whole tissue sections from patients with node-negative IDC who did not receive adjuvant systemic therapy (*n *= 233). Nuclear Stat5a (Nuc-Stat5a) scores were calculated through pathologist scoring of traditional DAB-IHC and obtained for 223 tumor specimens.

Material IV was a breast cancer tissue microarray (0.6-mm cores) from Yale University pathology archives, representing 291 node-negative IDC patients who did not receive adjuvant systemic therapy [30]. Nuc-Stat5a AQUA scores were obtained for 239 tumors.

Material V comprised whole tissue sections from tumors of node-negative IDC patients who received adjuvant hormone monotherapy (*n *= 75). Specimens were obtained through the National Cancer Institute Cooperative Breast Cancer Tissue Resource. Nuc-Stat5a DAB-IHC scores were obtained for 73 tumor specimens.

Material VI was a 0.6-mm tumor tissue core microarray from a random series of breast cancer patients identified through the Alberta Cancer Registry (Calgary, Alberta, Canada) who received adjuvant hormone monotherapy for up to 60 months (average, 30 months) [30]. The array was constructed in triplicate from tumors of 50 patients who died of breast cancer and 50 patients with greater than 5-year follow-up without breast cancer recurrence. AQUA analysis yielded informative data on Nuc-Stat5a for 71 cases. Stat5a and Stat5b mRNA expression levels were evaluated in the context of clinical outcome in RNA microarray datasets (Material II) compiled from public repositories, Gene Expression Omnibus [36] and ArrayExpress [37], as described previously [38].

### Prolactin-induced *Stat5a *and *Stat5b *gene profiles

Adenoviral delivery of Stat5a and Stat5b was performed as previously described [21,39]. In brief, confluent MCF7 cells were infected with adenovirus in serum-free media (1 × 10^6 ^cells/well; multiplicity of infection (MOI) = 40) for 90 minutes at 37°C and cultured in regular growth media for 24 hours. Cells were serum starved for 16 hours before treatment with or without 10 n*M *PRL for 20 minutes (protein detection) or 4 hours (mRNA analysis). Stat5a and Stat5b expression and phosphorylation were determined by standard immunoblotting. Genome-wide transcript profiling of mRNA (RNeasy Kit; Qiagen Inc., Valencia, CA, USA) was performed in triplicate by the KCC Genomics Core. RNA was validated by using the Agilent Bioanalyzer (Santa Clara, CA, USA) followed by cDNA synthesis and labeling for microarray expression profiling by using the HuGene 1.0 ST Array (Affymetrix, Santa Clara, CA, USA; GEO: GSE37781 [40]).

### Quantification of Stat5a and Stat5b by automated quantitative analysis (AQUA)

Rabbit polyclonal antisera specific for Stat5a and Stat5b were generated against unique epitopes of each protein, as previously described [5]. Antibody specificity was verified by cross-immunoprecipitation followed by immunoblotting and by immunohistochemistry on formalin-fixed, paraffin-embedded breast tissues in the presence or absence of blocking immunizing peptide. Immunofluorescent staining of Stat5a or Stat5b for AQUA analysis was performed on a Dako Autostainer by following a reported protocol [30] with Stat5a (1:8,000) or Stat5b (1:4,000) antisera used for primary detection. The AQUA/PM2000 platform (HistoRx, New Haven, CT, USA) [33] was used as previously described [22,30] to quantify cellular and nuclear levels of Stat5a or Stat5b (Cy5) within tumor tissue, as defined by cytokeratin-positive (FITC/Alexa-488) and nuclei-positive (DAPI) mapping. Cases not interpretable by AQUA because of loss of histospots or insufficient staining quality or tumor sampling were excluded [33].

### Detection of Stat5a by DAB-chromogen immunohistochemistry

DAB chromogen immunohistochemistry (DAB-IHC) for Stat5a and Stat5b was performed by following the previously described protocol by using a Dako Autostainer Plus (Dako, Carpinteria, CA, USA) with the following modifications [29,30]. Antigen retrieval was performed by using the DAKO PT-module with citric acid buffer (pH 6.0). Stat5a (1:8,000) or Stat5b (1:4,000) antibody was incubated with tissue slides for 30 minutes at room temperature. Transcriptionally active Stat5a, defined by nuclear localization of the protein (Nuc-Stat5a), was identified by a pathologist blinded to clinical outcome. Control experiments validating nuclear localization of the Stat5a protein in response to prolactin were performed by using viable surgically removed healthy human breast tissues obtained from Thomas Jefferson University Hospital under IRB-approved protocols. Explants were cultured *ex vivo *at 37°C in RPMI media containing 10% FBS and 1 m*M *sodium pyruvate, with or with 100 n*M *human prolactin for 1 hour and subsequently formalin fixed, paraffin embedded, and sectioned. Standard DAB-IHC was performed as described previously [29,30].

### Statistical methods

Affymetrix HuGene ST 1.0 expression-signal estimates were computed by using iterPLIER in Affymetrix Expression Console version 1.1. Genes represented by multiple probesets were averaged, and expression values were scaled to the probeset with the maximal variance for that gene. Low-signal genes were filtered out by retaining genes expressed above the 25th percentile on at least one array. Approximately 85% of genes were retained and evaluated for differential expression between Stat5a or Stat5b-expressing cells in the presence or absence of prolactin by using Significance Analysis for Microarrays with 25% false discovery rate and 1.25 minimum fold-change cutoffs.

One-way ANOVA with Dunnett T3 pairwise *post hoc *test (SPSS v15.0; SPSS Inc, Chicago, IL, USA) was used to compare levels of Stat5a or Stat5b between breast histology groups in progression Material I. Clinical end points for survival analyses in Materials III, V, and VI were breast cancer-specific survival (CSS) and time-to-recurrence (TTR) of either local or distant disease, according to consensus definitions [41]. For Material IV, only CSS was available. Statistical software R (v2.11.1) [42] determined optimal cutpoints for low and high Stat5a-expressing tumors as a function of patient survival in all materials. Survival analyses were conducted by using Kaplan-Meier plots, log-rank test, and adjusted Cox regression when proportional hazard assumption passed (assessed globally by using a Wald goodness-of-fit χ^2 ^test for each cohort and outcome multivariate model) or adjusted Weibull regression when proportional hazard assumption failed (SAS v9.2; SAS Institute, Cary, NC, USA). When available, variables included in the adjusted models were tumor grade, tumor size, lymph node status, ER/PR expression, Her2 status, and Nuc-Stat5a.

## Results

### Levels of nuclear-localized Stat5a but not Stat5b are diminished during breast cancer progression

**Figure 1:**
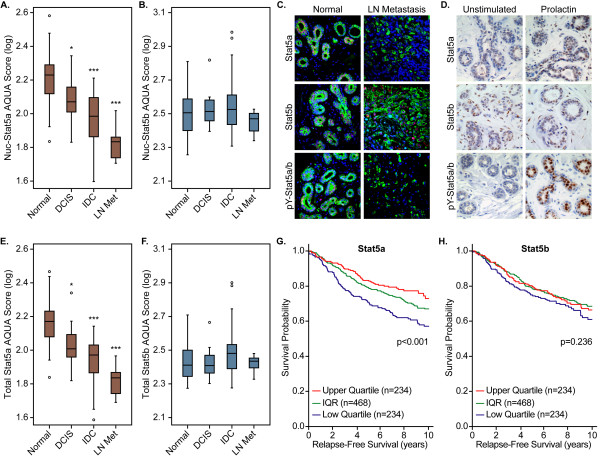
**Nuclear localization of Stat5a (Nuc-Stat5a) is lost during breast cancer progression**. **(A) **Detection of nuclear-localized Stat5a in Material I with immunofluorescence and quantified with AQUA revealed a significant reduction in Nuc-Stat5a protein in invasive ductal carcinoma (IDC; *n *= 66) and lymph node metastases (*n *= 19) when compared with normal breast tissue (*n *= 23) and ductal carcinoma *in situ *(DCIS; *n *= 18). **(B) **Levels of nuclear localized Stat5b (Nuc-Stat5b) remained unchanged between normal (*n *= 24), DCIS (*n *= 12), IDC (*n *= 67), and lymph node metastases (*n *= 13) in the same breast progression array (Material I). **(C) **Representative images of Stat5a, Stat5b, and pY-Stat5a/b detected with immunofluorescence in normal human breast tissue and lymph node metastases (Material I). Stat5a, Stat5b, pY-Stat5a/b, red (Cy5); cytokeratin, green (FITC); nuclei, blue (DAPI). **(D) **Stat5a protein (upper panels) translocated to the nucleus after *ex vivo *prolactin stimulation of human breast tissue explants, whereas prolactin induced only minimal nuclear localization of Stat5b protein (middle panels). Corresponding with enrichment of nuclear Stat5a protein in response to prolactin, tyrosine-phosphorylated Stat5a/b (pY-Stat5a/b) was induced in the nuclei of the same breast tissue after *ex vivo *stimulation with prolactin (lower panels). **(E, F) **Levels of total cellular Stat5a protein (E), but not total cellular Stat5b protein (F), were significantly reduced over breast cancer progression (Material I). **(G, H) **Stat5a mRNA expression levels in human breast tumor tissue (Material II) were associated with relapse-free survival (G), whereas Stat5b mRNA expression levels were not associated with relapse-free survival (H). Statistical differences (ANOVA, Dunnett T3 *post hoc *test) in levels of nuclear and total cellular Stat5a or Stat5b over breast cancer progression in relation to normal histologic type are indicated. ***p < 0.001; *p < 0.05. AQUA, Automated Quantitative Analysis; DCIS, ductal carcinoma *in situ*; IDC, invasive ductal carcinoma; IQR, interquartile range; LN Met, lymph node metastasis.

To investigate whether previously reported reduced levels of Nuc-pYStat5a/b during breast cancer progression could in part be caused by loss of Stat5a or Stat5b protein expression, a breast tissue progression array (Material I) that included normal breast, ductal carcinoma *in situ *(DCIS), invasive ductal carcinoma (IDC), and lymph node metastases, was stained for Stat5a or Stat5b with immunofluorescence labeling and quantified with AQUA (Figure 1). Levels of Nuc-Stat5a were markedly reduced during breast cancer progression, with significant loss of Nuc-Stat5a expression in IDC (*P *< 0.001) and greatest loss in lymph node metastases (*P *< 0.001; Figure 1A, C). Interestingly, loss of Nuc-Stat5a over progression occurred in parallel with our previously reported loss of Nuc-pYStat5a/b in the same breast-progression tissue array (Figure 1C) [30]. In contrast, Nuc-Stat5b remained detectable, and levels did not change over progression (Figure 1B, C). These quantitative *in situ *data provided novel information indicating that levels of Nuc-Stat5a, but not Nuc-Stat5b, are reduced over breast cancer progression.

To verify lack of cross-reactivity between the Stat5a and Stat5b polyclonal antibodies developed to the unique C-termini of Stat5a and Stat5b [5], the two proteins were individually immunoprecipitated from SKBR3 breast cancer cell lysates and resolved by gel electrophoresis. Cross-immunoblotting with the same Stat5a-specific or Stat5b-specific antibodies demonstrated specificity of each antibody (see Additional File 1, panel A). Immunoblotting Stat5a or Stat5b immunoprecipitations with a pan-Stat5a/b antibody further confirmed specificity by detecting only single bands in each immunoprecipitation corresponding to the slightly slower-migrating Stat5a band (94 kDa) or the slightly faster migrating Stat5b band (92 KDa). Antibody specificity for detection of Stat5a or Stat5b was further extended to immunohistochemistry on formalin-fixed, paraffin-embedded tissue in the presence or absence of blocking peptides representing the unique immunogens.

Detection of Stat5a or Stat5b was reduced in peptide-competed conditions compared with control conditions (see Additional File 1, panel B). In the absence of selective phospho-tyrosine-Stat5a or phospho-tyrosine-Stat5b antibodies, levels of nuclear Stat5a or nuclear Stat5b can be measured as a proxy for tyrosine-phosphorylated, nuclear-localized and transcriptionally active protein. After *ex vivo *prolactin stimulation of viable human breast tissue explants, prolactin preferentially increased levels of Stat5a protein in the nucleus of normal luminal epithelial cells, but was less effective at enhancing nuclear levels of Stat5b protein (Figure 1D). Corresponding to enhanced levels of nuclear Stat5a in response to prolactin, tyrosine-phosphorylated Stat5a/b was highly enriched in the epithelial cell nuclei of the same prolactin-stimulated tissues (Figure 1D).

### Total cellular expression of Stat5a but not Stat5b protein is suppressed over breast cancer progression

The reduced levels of nuclear-localized Stat5a protein in invasive breast cancer and lymph node metastases are consistent with our previous reports showing a loss of Nuc-pYStat5a/b [29,30]. We previously showed that levels of the Jak2 phosphatase, PTP1B, were inversely correlated with Nuc-pYStat5a/b levels in human breast cancer and that PTP1B suppressed prolactin-induced Stat5a phosphorylation levels [43], suggesting that low levels of Nuc-pYStat5a/b in breast cancer may in part be due to reduced phosphorylation of Stat5a. To determine whether the observed reduction in Nuc-Stat5a levels could be attributed to a reduction of total cellular Stat5a protein, we quantified levels of total cellular Stat5a protein by using AQUA within the breast tissue progression array. Interestingly, total cellular levels of Stat5a protein were significantly reduced from normal epithelia to invasive and metastatic breast cancer (*P *< 0.001; Figure 1E). In contrast, total cellular Stat5b levels remained unchanged across the progression material (Figure 1F). We conclude that loss of Nuc-Stat5a in invasive breast cancer and metastases reflects, in part, loss of total cellular expression of Stat5a protein and is not simply a result of reduced tyrosine phosphorylation and nuclear translocation or due to increased nuclear exclusion of Stat5a.

### mRNA levels for Stat5a but not Stat5b are prognostic of breast cancer relapse

Tumor levels of Stat5a and Stat5b mRNA in a cohort of 936 patients with available outcome data were then interrogated to determine whether expression of Stat5a or Stat5b in primary invasive breast cancer was associated with clinical outcome. Interestingly, patients whose tumors were in the lowest quartile of Stat5a mRNA expression levels were associated with reduced time to breast cancer relapse, whereas the highest quartile of Stat5a mRNA expression levels associated with longer time to relapse (Figure 1G). However, Stat5b mRNA levels were not informative about the risk of breast cancer relapse (Figure 1H).

### Stat5a and Stat5b modulate different gene-transcription profiles in breast cancer

Despite high homology, transcript profiles were shown to differ significantly after selective knockdown of either Stat5a or Stat5b expression by using siRNA in BaF3 cells, a murine pro-B cell line [44]. In MCF-7 human breast cancer cells, expression of selected genes also differed after stable introduction of constitutively active Stat5a or Stat5b [19].

More directly and in an unbiased manner to determine the degree of overlap of immediate-early transcripts modulated by Stat5a or Stat5b in MCF-7 cells, we performed genome-wide transcript profiling after transient overexpression of Stat5a or Stat5b by using adenoviral gene delivery, followed by a brief 4-hour exposure to human prolactin. This experimental strategy avoids basal activation, because in these cells, exogenous wild-type Stat5a or Stat5b remains unphosphorylated until prolactin stimulation (see Additional File 2). Consistent with divergent target genes for Stat5a and Stat5b, genome-wide transcript profiling revealed that less than 10% of the top 150 modulated genes were common (see Additional File 3).

### Nuclear localized Stat5a is an independent marker of prognosis in node-negative breast cancer

**Figure 2:**
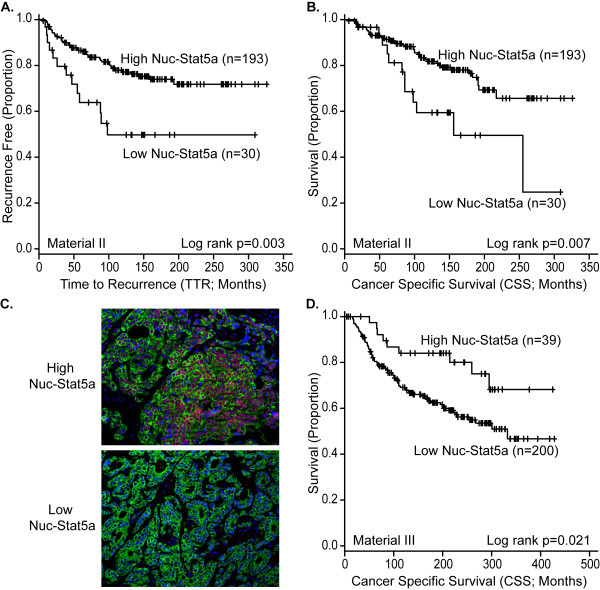
**Loss of nuclear Stat5a (Nuc-Stat5a) predicts unfavorable breast cancer prognosis**. **(A, B) **Nuclear localization of Stat5a was detected with standard DAB IHC and pathologist review of whole-tissue sections in Material III. Kaplan-Meier analysis indicated that patients with low levels of Nuc-Stat5a had **(A) **reduced time-to-recurrence (TTR) of breast cancer and (B) poor breast cancer-specific survival (CSS). **(C, D) **Nuclear localization of immunofluorescently labeled Stat5a was quantified by AQUA analysis in a tissue microarray of node-negative breast cancer. (C) Representative immunofluorescent images of high and low Stat5a expression in invasive ductal carcinoma. (D) Quantitation of nuclear levels of Stat5a by AQUA revealed by Kaplan-Meier analysis that loss of Nuc-Stat5a is prognostic of poor breast cancer-specific survival (CSS) in Material IV. Censored cases (+) and number of patients per group are indicated.

Based on our observations that Stat5a but not Stat5b mRNA levels correlated with breast cancer outcome and that Stat5a protein is selectively reduced over breast cancer progression, we evaluated Stat5a protein levels with immunohistochemistry in formalin-fixed, paraffin-embedded archival breast tumor tissues with clinical outcome. We first evaluated expression of Nuc-Stat5a by using DAB-IHC on whole tissue sections in node-negative breast cancer from patients who did not receive any systemic adjuvant treatment (Material III). Survival analyses evaluated time to recurrence (TTR) and cancer-specific survival (CSS). Low levels of Nuc-Stat5a were associated with increased risk of breast cancer recurrence (TTR; log-rank, *P *= 0.003; *n *= 223; Figure 2A; univariate Weibull regression hazard ratio (HR) = 2.60 (1.36, 4.96), *P *= 0.004; *n *= 218, see Additional File 4). Importantly, low expression of Nuc-Stat5a also was associated with poor breast cancer-specific survival (CSS; log-rank *P *= 0.007; *n *= 223, Figure 2B; univariate Weibull regression HR = 2.35 (1.20, 4.58); *P *= 0.012; *n *= 218; Table 2). To validate these data, we analyzed levels of Nuc-Stat5a with immunofluorescence and quantified expression with AQUA in a tissue microarray from an independent cohort of adjuvant therapy-naïve, node-negative breast cancer patients (Material IV). Low levels of Nuc-Stat5a (Figure 2C) predicted poor breast cancer survival in Material IV (CSS; log-rank *P *= 0.021; *n *= 239, Figure 2D; univariate Weibull regression HR = 2.13 (1.01, 4.49), *P *= 0.047; *n *= 190, Table 2). TTR data were not available for Material IV.

**Table 2:** Univariate and multivariate survival analyses of breast cancer-specific survival (CSS) in Materials III and IV

Material III CSS (*n *= 218)			Multivariate adjusted (Weibull)		Univariate unadjusted (Weibull)	
Variable		*n*	Hazard ratio (95% CI)	*P *value	Hazard ratio (95% CI)	*P *value
Grade	1	57	1	-	1	-
	2	95	1.01 (0.45 to 2.26)	0.977	1.33 (0.62 to 2.85)	0.472
	3	66	1.59 (0.65 to 3.87)	0.306	1.90 (0.88 to 4.08)	0.102

Size	< 2 cm	108	1	-	1	-
	2 to ≥ 5 cm	101	1.59 (0.82 to 3.08)	0.166	1.88 (1.03 to 3.45)	0.041
	> 5 cm	9	2.38 (0.74 to 7.60)	0.145	2.86 (0.94 to 8.76)	0.065

ER/PR status	Neg	43	1	-	1	-
	Pos	175	1.49 (0.68 to 3.26)	0.321	0.87 (0.45 to 1.70)	0.688

Stat5a	Low (0)	30	2.34 (1.16 to 4.71)	0.018	2.35 (1.20 to 4.58)	0.012
	High (> 0)	188	1	-	1	-

Global test for PH assumption: χ^2 ^(4) = 17.18; *P *= 0.0018.						

**Material IV CSS (*n *= 190)**			**Multivariate adjusted (Weibull)**		**Univariate unadjusted (Weibull)**	
**Variable**		***n***	**Hazard ratio (95% CI)**	***P *value**	**Hazard ratio (95% CI)**	***P *value**

Grade	1	42	1	-	1	-
	2	110	1.55 (0.84 to 2.84)	0.162	1.42 (0.77 to 2.59)	0.258
	3	38	1.11 (0.50 to 2.47)	0.797	1.10 (0.52 to 2.35)	0.801

Size	< 2 cm	71	1	-	1	-
	2 to ≥5 cm	98	2.10 (1.19 to 3.72)	0.011	2.01 (1.14 to 3.55)	0.016
	≥5 cm	21	3.48 (1.61 to 7.54)	0.002	3.09 (1.45 to 6.58)	0.004

ER/PR status	Neg	41	1	-	1	-
	Pos	149	0.93 (0.49 to 1.75)	0.824	0.91 (0.52 to 1.59)	0.738

Her2 status	Neg	166	1	-	1	-
	Pos	24	1.02 (0.47 to 2.20)	0.963	0.97 (0.48 to 1.96)	0.939

Stat5a	Low (< 881)	158	2.15 (1.02 to 4.55)	0.045	2.13 (1.01 to 4.49)	0.047
	High (≥881)	32	1	-	1	-

Global test for PH assumption: χ^2^(5) = 10.05; *P *= 0.074.

Nuc-Stat5a remained an independent marker of disease prognosis in multivariate analyses in both Materials III and IV. When adjusting for standard clinical and pathologic markers in Material III, patients with low Nuc-Stat5a had an adjusted 2.5-fold increased risk of disease recurrence (TTR; multivariate Weibull regression HR = 2.55 (1.31, 4.98), *P *= 0.006; *n *= 218, Additional File 4) and a 2.3-fold greater risk of dying of breast cancer (CSS; multivariate Weibull regression HR = 2.34 (1.16, 4.71), *P *= 0.018; *n *= 218; Table 2). Likewise, in Material IV, AQUA-quantified Nuc-Stat5a was an independent prognostic indicator of breast cancer-specific survival, as reflected in a 2.2-fold increased risk of death (CSS; multivariate Weibull regression HR = 2.15 (1.02, 4.55), *P *= 0.045; *n *= 190; Table 2). We found comparable effect sizes in two separate cohorts and by two analytic approaches, and conclude that Nuc-Stat5a is an independent prognostic marker of outcome in patients with lymph node-negative breast cancer.

When patients who received radiation therapy were excluded from these analyses, smaller sample sizes limited the power, but effect sizes associated with Nuc-Stat5a were unchanged. Further, Nuc-Stat5a remained an independent marker of prognosis when radiation therapy was included in multivariate analyses (data not shown).

### Loss of nuclear Stat5a is associated with unfavorable outcome in antiestrogen-treated breast cancer patients

**Figure 3:**
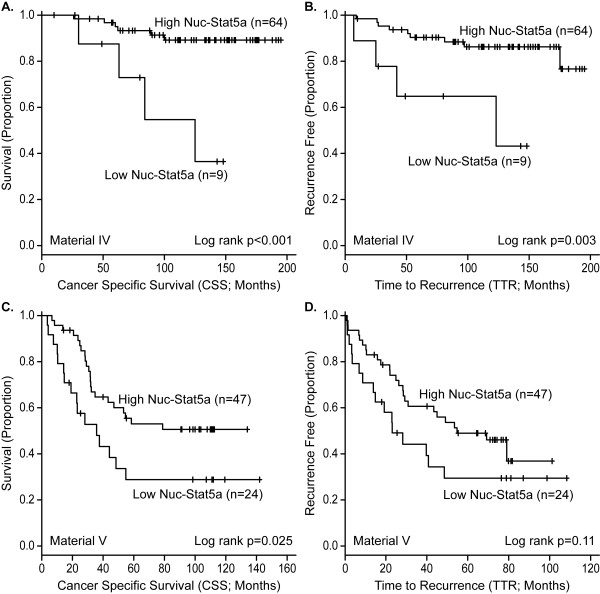
**Low levels of nuclear Stat5a (Nuc-Stat5a) predict poor response to antiestrogen therapy**. **(A, B) **Nuc-Stat5a was detected by DAB-chromogen IHC and pathologist scoring of whole-tissue sections from node-negative breast cancer patients treated with antiestrogen monotherapy (Material V). The lack of nuclear localization of Stat5a predicted (A) poor breast cancer-specific survival (CSS) and (B) reduced time-to-recurrence (TTR) of breast cancer. **(C, D) **Nuc-Stat5a expression levels were measured with immunofluorescence and quantified with AQUA in node-negative and -positive breast cancer patients treated with antiestrogen monotherapy (Material VI). Low levels of Nuc-Stat5a were predictive of (C) poor cancer-specific survival (CSS) and (D) reduced TTR of breast cancer. Kaplan-Meier plots with censored cases (+) and number of patients per group indicated.

To determine whether Nuc-Stat5a may be a predictor of outcome in patients treated with antiestrogen therapy, we first analyzed Nuc-Stat5a expression levels by DAB-IHC and pathologist scoring of whole tissue sections from a cohort of node-negative breast cancer patients who received adjuvant antiestrogens and did not receive chemotherapy (Material V). The absence of detectable Nuc-Stat5a in these tumors indicated a significantly increased risk of breast cancer-specific death (CSS; log-rank *P *< 0.001; *n *= 73; Figure 3A; univariate Cox regression HR = 6.73 (1.88, 24.05); *P *= 0.003; *n *= 73). Patients with undetectable Nuc-Stat5a were also at an increased risk of breast cancer recurrence (TTR; log-rank *P *= 0.003; *n *= 73; Figure 3B; univariate Cox regression HR = 5.08 (1.52, 17.01); *P *= 0.008; *n *= 73). Multivariate analysis of this cohort revealed that Nuc-Stat5a remained an independent marker of patient outcome after adjustment for other tumor parameters both by CSS (multivariate Cox regression HR = 4.19 (1.13, 15.48); *P *= 0.032; *n *= 73) and by TTR (multivariate Cox regression HR = 4.27 (1.20, 15.19); *P *= 0.025; *n *= 73). ER/PR status was not included in the multivariate model because only one tumor was ER/PR negative.

Analysis of Nuc-Stat5a expression as a function of survival was then performed in an independent tissue microarray of tumors, which included those from both node-negative and node-positive patients who were also treated exclusively with antiestrogen monotherapy (Material VI). Nuc-Stat5a was detected with immunofluorescence and quantified with AQUA. Patients whose tumors expressed low levels of Nuc-Stat5a were at markedly increased risk of antiestrogen treatment failure in this independent material. Risk of death of breast cancer was significantly increased in patients with low levels of Nuc-Stat5a (CSS log-rank *P *= 0.025; *n *= 71; Figure 3C; univariate Cox regression HR = 2.75 (1.33, 5.69); *P *= 0.006; *n *= 55; Table 3). Univariate Cox regression also revealed a significant increased risk of breast cancer recurrence in these patients (TTR univariate Cox regression HR = 2.15 (1.06, 4.35); *P *= 0.033; *n *= 55), although despite a clear separation of Kaplan-Meier curves, log-rank analysis did not reach statistical significance (TTR log-rank *P *= 0.11; *n *= 71; Figure 3D). In multivariate analysis adjusting for tumor size, tumor grade, lymph node status, ER/PR status, and Her2 overexpression, Nuc-Stat5a remained an independent marker of survival in Material VI, predicting a fivefold increased risk of breast cancer-related death (CSS; multivariate Cox regression HR = 4.95 (1.87, 13.06); *P *= 0.001; *n *= 55; Table 3). Positive node status (CSS; multivariate Cox regression HR = 4.72 (1.90, 11.74); *P *< 0.001; *n *= 55) and tumor size (CSS; multivariate Cox regression tumor size 2 to < 5 cm; HR = 3.51 (1.26, 9.74); *P *= 0.016 and tumor size ≥5 cm; HR = 8.54 (1.92, 37.94); *P *= 0.005; *n *= 55) were also independent predictors of survival (Table 3). Although tumors with low Nuc-Stat5a trended toward earlier breast cancer recurrence, TTR did not reach statistical significance in multivariate analysis of Material VI (TTR; multivariate Cox regression HR = 2.14 (0.89, 5.12); *P *= 0.087; *n *= 55). Collectively, based on two independent cohorts of patients treated with antiestrogen therapy alone, low levels of Nuc-Stat5a were in both cohorts associated with elevated risk of failure of antiestrogen treatment. These initial studies justify further analysis of Nuc-Stat5a as a potentially clinically useful measurement of response to antiestrogen therapy.

**Table 3:** Univariate and multivariate Cox regression survival analysis of breast cancer-specific survival (CSS) and time to recurrence (TTR) as a function of Nuc-Stat5a in breast cancer patients treated with antiestrogen monotherapy (Materials V and VI)

Material V CSS (*n *= 73)			Multivariate adjusted (Cox)		Univariate unadjusted (Cox)	
Variable		*n*	Hazard ratio (95% CI)	*P *value	Hazard ratio (95% CI)	*P *value
Grade	1	17	1	-	1	-
	2	43	3.42 (0.42 to 27.87)	0.251	3.74 (0.46 to 30.52)	0.218
	3	13	2.60 (0.23 to 29.30)	0.438	3.32 (0.30 to 36.66)	0.327

Size	< 2 cm	40	1	-	1	-
	≥2 to ≥5 cm	33	4.29 (0.86 to 21.38)	0.075	5.79 (1.23 to 27.26)	0.026

Stat5a	Low (0)	9	4.19 (1.13 to 15.48)	0.032	6.73 (1.88 to 24.05)	0.003
	High (> 0)	64	1	-	1	-

Global test for PH assumption: χ^2^(3) = 1.67; *P *= 0.64.						

**Material V TTR (*n *= 73)**			**Multivariate adjusted (Cox)**		**Univariate unadjusted (Cox)**	
**Variable**		***n***	**Hazard ratio (95% CI)**	***P *value**	**Hazard ratio (95% CI)**	***P *value**

Grade	1	17	1		1	
	2	43	1.20 (0.30 to 4.81)	0.794	1.27 (0.32 to 5.04)	0.729
	3	13	1.59 (0.31 to 8.22)	0.581	1.87 (0.37 to 9.39)	0.448

Size	< 2 cm	40	1		1	
	≥2 to ≥5 cm	33	1.63 (0.49 to 5.43)	0.429	2.24 (0.73 to 6.86)	0.158

Stat5a	Low (0)	9	4.27 (1.20 to 15.19)	0.025	5.08 (1.52 to 17.01)	0.008
	High (> 0)	64	1		1	

Global test for PH assumption: χ^2^(3) = 1.47; *P *= 0.69.						

**Material VI CSS (*n *= 55)**			**Multivariate adjusted (Cox)**		**Univariate unadjusted (Cox)**	
**Variable**		***n***	**Hazard ratio (95% CI)**	***P *value**	**Hazard ratio (95% CI)**	***P *value**

Grade	1	8	1	-	1	-
	2	28	3.48 (0.91 to 13.31)	0.068	1.11 (0.36 to 3.41)	0.855
	3	19	2.83 (0.65 to 12.28)	0.164	2.25 (0.73 to 6.91)	0.158

Size	< 2 cm	24	1	-	1	-
	2 to ≥5 cm	26	3.51 (1.26 to 9.74)	0.016	4.22 (1.76 to 10.13)	0.001
	≥5 cm	5	8.54 (1.92 to 37.94)	0.005	6.94 (2.01 to 24.00)	0.002

LN status	Neg	29	1	-	1	-
	Pos	26	4.72 (1.90 to 11.74)	< 0.001	4.30 (1.98 to 9.33)	< 0.001

ER/PR status	Neg	8	1	-	1	-
	Pos	47	0.73 (0.22 to 2.38)	0.597	0.37 (0.16 to 0.86)	0.021

Her2 status	Neg	48	1	-	1	-
	Pos	7	2.87 (0.92 to 8.96)	0.070	3.07 (1.23 to 7.63)	0.016

Stat5a	Low (< 1,454)	16	4.95 (1.87 to 13.06)	0.001	2.75 (1.33 to 5.69)	0.006
	High (≥1,454)	39	1	-	1	-

Global test for PH assumption: χ^2^(6) = 3.19; *P *= 0.78.						

**Material VI TTR (*n *= 55)**			**Multivariate adjusted (Cox)**		**Univariate unadjusted (Cox)**	
**Variable**		***n***	**Hazard ratio (95% CI)**	***P *value**	**Hazard ratio (95% CI)**	***P *value**

Grade	1	8	1	-	1	-
	2	28	1.17 (0.37 to 3.72)	0.794	0.87 (0.31 to 2.41)	0.783
	3	19	1.24 (0.33 to 4.68)	0.746	1.98 (0.71 to 5.55)	0.191

Size	< 2 cm	24	1	-	1	-
	2 to ≥5 cm	26	3.38 (1.30 to 8.80)	0.013	3.75 (1.67 to 8.43)	0.001
	≥5 cm	5	8.17 (2.26 to 29.50)	0.001	6.91 (2.27 to 21.06)	< 0.001

LN status	Neg	29	1	-	1	-
	Pos	26	3.46 (1.57 to 7.64)	0.002	3.42 (1.67 to 7.01)	< 0.001

ER/PR status	Neg	8	1	-	1	-
	Pos	47	0.51 (0.17 to 1.48)	0.214	0.30 (0.13 to 0.68)	0.004

Her2 status	Neg	48	1	-	1	-
	Pos	7	3.35 (1.13 to 9.93)	0.029	3.06 (1.24 to 7.56)	0.015

Stat5a	Low (< 1,454)	16	2.14 (0.89 to 5.12)	0.087	2.15 (1.06 to 4.35)	0.033
	High (≥1,454)	39	1	-	1	-

Global test for PH assumption: χ^2^(6) = 4.67; *P *= 0.59. CI, confidence interval; ER/PR status, estrogen or progesterone receptor positive; HR, hazard ratio; LN status, lymph node status.

## Discussion

The present study used quantitative *in situ *analysis to reveal for the first time a significant reduction in total cellular and nuclear Stat5a protein levels in invasive breast cancer and lymph node metastases compared with normal breast epithelia and DCIS. In contrast, Stat5b protein levels remained unchanged, suggesting divergent expression and involvement of Stat5a and Stat5b during breast cancer progression. Consistent with the notion of distinct roles in human breast cancer, experimental hyperactivation of transcription factors Stat5a or Stat5b independently in MCF-7 breast cancer cells revealed only limited overlap between Stat5a- and Stat5b-responsive genes. Likewise, low mRNA expression for Stat5a but not Stat5b correlated with poor prognosis in a cohort of more than 900 patients. *In situ *analysis of two separate cohorts totaling more than 500 patients with therapy-naïve, lymph node-negative breast cancer identified low levels of nuclear localized Stat5a (Nuc-Stat5a) protein as an independent marker of poor prognosis. In both of these patient cohorts, reduced levels of Nuc-Stat5a protein were associated with greater than twofold increased risk of death of breast cancer after adjustment for other clinical parameters in multivariate analyses.

Analysis of tumors from a second pair of independent cohorts of patients, who had received adjuvant antiestrogen monotherapy, identified loss of Nuc-Stat5a protein as an independent marker associated with greater than fourfold increased risk of death from breast cancer in this population.

A general shortcoming of previous studies of Stat5 expression in breast cancer has been the lack of analytic distinction of Stat5a from the highly homologous Stat5b. Our novel observations suggest a selective loss of Stat5a protein during breast cancer progression, representing a newly defined mechanism to explain partially the observed frequent loss of Nuc-pYStat5a/b during breast cancer progression [29,30]. Alternative but not mutually exclusive mechanisms recently reported include disruption of Stat5a/b phosphorylation during breast cancer progression through upregulation of the Jak2 tyrosine phosphatase, PTP1B [43], or inhibitory signaling to Stat5 by the truncated ERBB2 isoform, p100-t-ERBB2 [45]. Importantly, loss of Stat5a protein may also be a consequence of reduced Stat5a activation because numerous putative Stat5a response elements occur within the *Stat5a *gene promoter [46], a possibility that will be explored in future studies.

The favorable prognosis associated with continued expression and nuclear localization of Stat5a in invasive breast cancer supports the hypothesis drawn from *in vitro *studies that, despite a potential role in tumor initiation, Stat5a signaling is important for maintaining tumor differentiation and suppressing disease progression in established human breast cancer [11,12,20,21]. Interestingly, the transcription factor NFAT1 exerts actions opposite to Stat5 in breast cancer by inhibiting tumor growth while promoting metastasis [47,48]. NFAT1 displayed reciprocal negative crosstalk with Stat5, and NFAT levels were inversely correlated with Stat5 levels in human breast cancer [49]. Additionally, the new outcome data help clarify previous immunohistochemical reports documenting prognostic and antiestrogen response-predictive associations of nuclear localized, tyrosine phosphorylated Stat5a/b (Nuc-pYStat5a/b) [29,30] or cellular Stat5 [31]. Considering that as many as one third of patients with ER-positive breast cancer develop resistance to antiestrogen therapy and relapse [50,51], identification of patients with increased risk of therapy failure for stratification into more aggressive therapies is also essential. Many strategies have been proposed to identify patients with therapy-resistant tumors, including PCR-based assays, gene-expression profiling, or immunohistochemical detection of biomarkers such as Her2, EGFR, IGF-IR, PR, p27, AIB1, IRS-1, caveolin-1, retinoblastoma tumor suppressor, and nuclear localized and tyrosine phosphorylated Stat5a/b [30,38,51-55]. Interestingly, Nuc-Stat5a remained an independent marker of prognosis and response to antiestrogen therapy when other putative markers of antiestrogen responsiveness, such as Her2 (Table 3), PR (Table 3) or Ki67 (data not shown), were included in multivariate analyses, and levels of Nuc-Stat5a did not correlate with Her2 or Ki67 expression (data not shown). Molecular mechanistic studies *in vivo *will be needed to determine whether causal mechanisms exist by which Stat5a affects tumor responsiveness to antiestrogen therapy.

Although the new data provide clarity about Stat5a and Stat5b in breast cancer, several limitations of this study exist. The present study focused on Stat5a and Stat5b expression in normal and malignant cells, but it will be important in future work to include analyses of expression of Stat5a and Stat5b in stromal tumor cells. A combination of quantitative analysis of fluorescent immunolabeling and standard pathologist-based scoring of DAB-chromogen staining was used. More robust data will come from future inclusion of additional cases and further standardized assay conditions, including development of assays for absolute quantification of Stat5a and Stat5b proteins in cellular compartments within the tumors, as has been achieved for other proteins [56]. Furthermore, optimal cutpoints for high and low Stat5a, derived by an objective method (R statistical software), differed between the cohorts, at least in part because of different analytic methods. However, clinically relevant Stat5a levels may differ between prognostic and antiestrogen therapy-response outcomes, as Stat5a may have distinct biologic functions relevant for each of the two clinical outcomes. Our cohorts were of limited size, and partial loss of evaluable tissues on the tissue microarrays and missing or incomplete clinical data for some of the parameters reduced the statistical power of some multivariate analyses. For instance, despite a strong predictive association of Nuc-Stat5a in CSS analyses in Material VI, TTR in Material VI failed to reach statistical significance by univariate log-rank (*P *= 0.11) and multivariate Cox regression models (*P *= 0.087), whereas univariate Cox regression in Material VI indicated a twofold significant increased risk of breast cancer recurrence (*P *= 0.033). This discrepancy could be a result of limited numbers of cases or reflect that data may not be missing at random, although specific patterns within missing data did not emerge within any cohort of patients examined in this study (data not shown).

It is perhaps surprising that tumor grade was not significant in univariate or multivariate analyses in the cohorts of this study, given evidence for prognostic association with histologic grade in breast cancer [57]. However, lack of detectable prognostic value of breast cancer grade is not uncommon [58,59] and may, at least in part, result from variable tissue handling and tumor grading between different pathologists, particularly because many of the tumor samples analyzed were obtained prior to the 1990s, when the importance of uniform grading systems became apparent and were adopted [57].

Finally, analyses were confined to retrospective cohorts and the antiestrogen predictive analyses must be followed up with studies of randomized prospective trial materials.

## Conclusions

Total Stat5a protein, as well as nuclear localization of Stat5a, is frequently lost in invasive breast cancer and lymph node metastases. Reduced levels of Nuc-Stat5a were associated with a more than twofold increased risk of unfavorable outcome in multivariate analyses of two independent cohorts of node-negative breast cancer, and may therefore represent a novel strong prognostic marker [60]. Furthermore, low levels of Nuc-Stat5a were associated with a more than fourfold increased risk of unfavorable outcome in multivariate analyses of two independent cohorts of patients treated with antiestrogen monotherapy. Low levels of Nuc-Sta5a therefore hold potential to become a new predictive marker of resistance to antiestrogen therapy, provided that the relation can be validated in clinical trial cohorts of patients randomized for antiestrogen therapy.

## Abbreviations

AQUA: Automated quantitative analysis; CI: confidence interval; CSS: breast cancer-specific survival; DAB-IHC: DAB-chromogen immunohistochemistry; DCIS: ductal carcinoma *in situ*; ER: estrogen receptor; FDR: false discovery rate; HR: hazard ratio; IDC: invasive ductal carcinoma; IQR: interquartile range; LN: lymph node; LN Met: lymph node metastasis; Nuc-pYStat5a/b: nuclear localized and tyrosine phosphorylated Stat5a/b; Nuc-Stat5a: nuclear localized Stat5a; Nuc-Stat5b: nuclear localized Stat5b; PR: progesterone receptor; SD: standard deviation; Stat5a: signal transducer and activator of transcription-5a; Stat5b: signal transducer and activator of transcription-5b; TTR: time to breast cancer recurrence.

## Competing interests

Dr. Rimm is a stockholder and paid consultant for HistoRx, the exclusive licensee of the Yale-held AQUA patent. Dr. Rimm receives royalties from the Yale-held AQUA patent. All other authors declare no competing interests.

## Authors' contributions

ARP, AKW, CDS, DLR, AMM, TH, and HR conceived the study and participated in its design. DLR, AMM, and HR provided formalin-fixed, paraffin-embedded archived patient materials for the study. ALR provided surgical tissue for *ex vivo *experiments. CL, THT, and MAG performed immunostaining, and AQUA. AKW, ACK, JAH, AJK, and CDS conducted pathologic reviews and evaluations. ARP, ACK, GAS, EP, BF, AE, and TH performed statistical analyses. AE conducted mRNA survival analyses. ARP conducted mRNA gene-analysis study. NY conducted *ex vivo *experiments. ARP and HR drafted the manuscript. All authors read and approved the final manuscript.

## Supplementary Material

Additional file 1 **Specificity of Stat5a and Stat5b polyclonal antibodies**. **(A) **Immunoprecipitation of Stat5a (94 kDa) or Stat5b (92 kDa) protein from SKBR3 breast cancer cells, followed by immunoblotting with the Stat5a, Stat5b, or a pan-Stat5a/b antibody, revealed specificity and lack of cross-reactivity between Stat5a and Stat5b antibodies. **(B) **Specificity of Stat5a and Stat5b antibodies in formalin-fixed, paraffin-embedded breast tissue was verified by using a blocking peptide assay. Antibodies were preincubated with the respective immunizing peptide or control before performing standard immunohistochemistry. Representative images from adjacent sections of the same tissues are shown. Click here for file

Additional file 2 **Adenoviral expression and prolactin-induced phosphorylation of Stat5a and Stat5b in MCF7 human breast cancer cells lines**. MCF7 cells were infected with adenovirus (MOI 40) expressing Stat5a or Stat5b and stimulated with prolactin for 20 minutes. Endogenous Stat5a and Stat5b were not detected with Western blot of whole-cell lysates from control cells, and phosphorylation of Stat5 was not detected in the absence of prolactin in control or Stat5a/Stat5b-overexpressing cells. Click here for file

Additional file 3 **Stat5a- and Stat5b-mediated genes**. List of 150 genes most significantly regulated by Stat5a or Stat5b in response to prolactin in MCF7 human breast cancer cell lines. *Genes modulated in common by Stat5a and Stat5b. FDR, false discovery rate. Click here for file

Additional file 4 **Univariate and multivariate Cox regression survival analyses of time to recurrence (TTR) of breast cancer in Material III**. CI, confidence interval; ER. estrogen receptor; HR, hazard ratio; PR, progesterone receptor. Click here for file
