# Supplementary figures and images for: Low levels of Stat5a protein in breast cancer are associated with tumor progression and unfavorable clinical outcomes
Source: Breast Cancer Res. 2012 Oct 4;14(5):R130. doi: 10.1186/bcr3328 (PMC4053108; doi:10.1186/bcr3328)

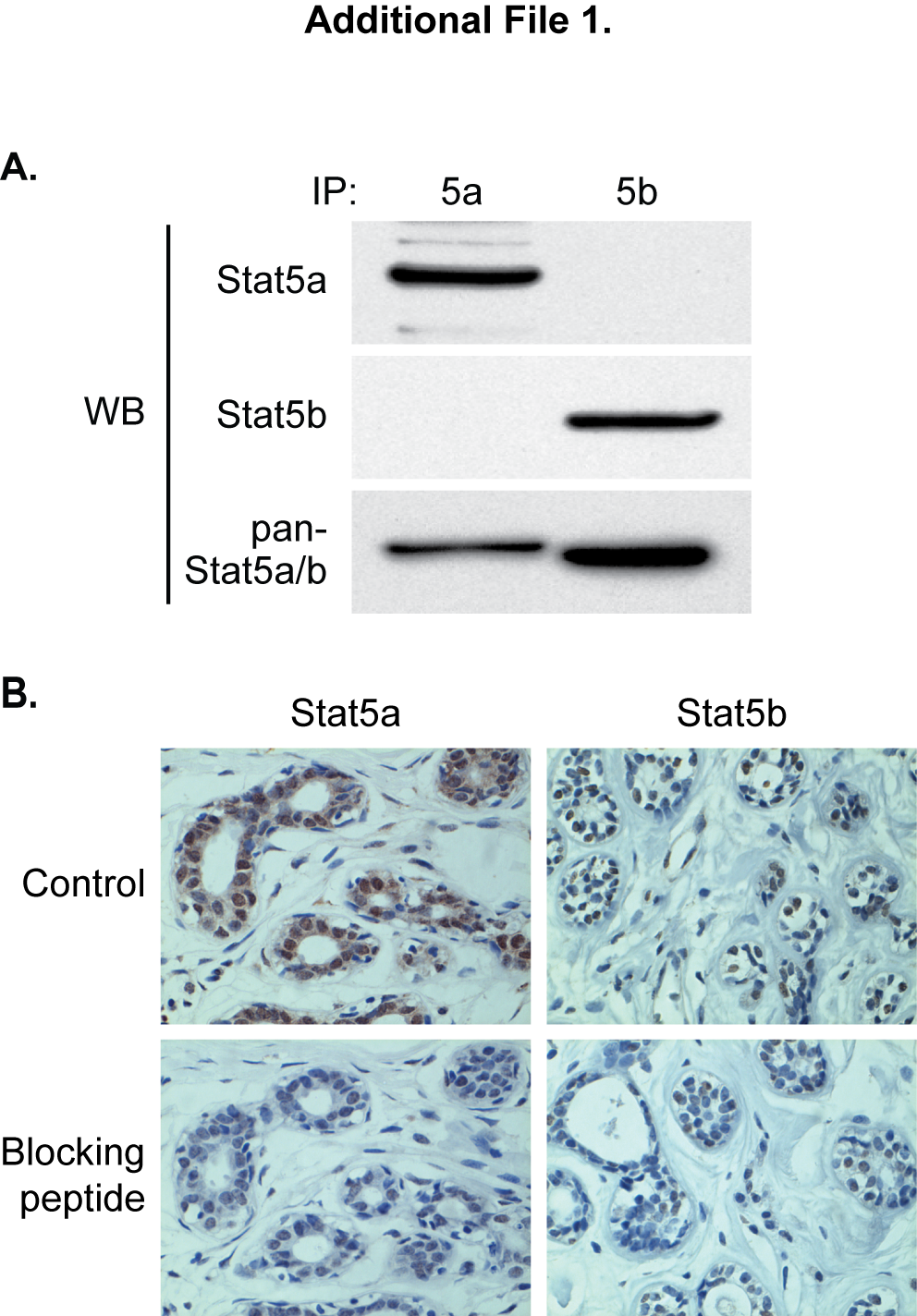

Supplement: Additional file 1 — Specificity of Stat5a and Stat5b polyclonal antibodies. (A) Immunoprecipitation of Stat5a (94 kDa) or Stat5b (92 kDa) protein from SKBR3 breast cancer cells, followed by immunoblotting with the Stat5a, Stat5b, or a pan-Stat5a/b antibody, revealed specificity and lack of cross-reactivity between Stat5a and Stat5b antibodies. (B) Specificity of Stat5a and Stat5b antibodies in formalin-fixed, paraffin-embedded breast tissue was verified by using a blocking peptide assay. Antibodies were preincubated with the respective immunizing peptide or control before performing standard immunohistochemistry. Representative images from adjacent sections of the same tissues are shown. [file bcr3328-S1.TIFF]

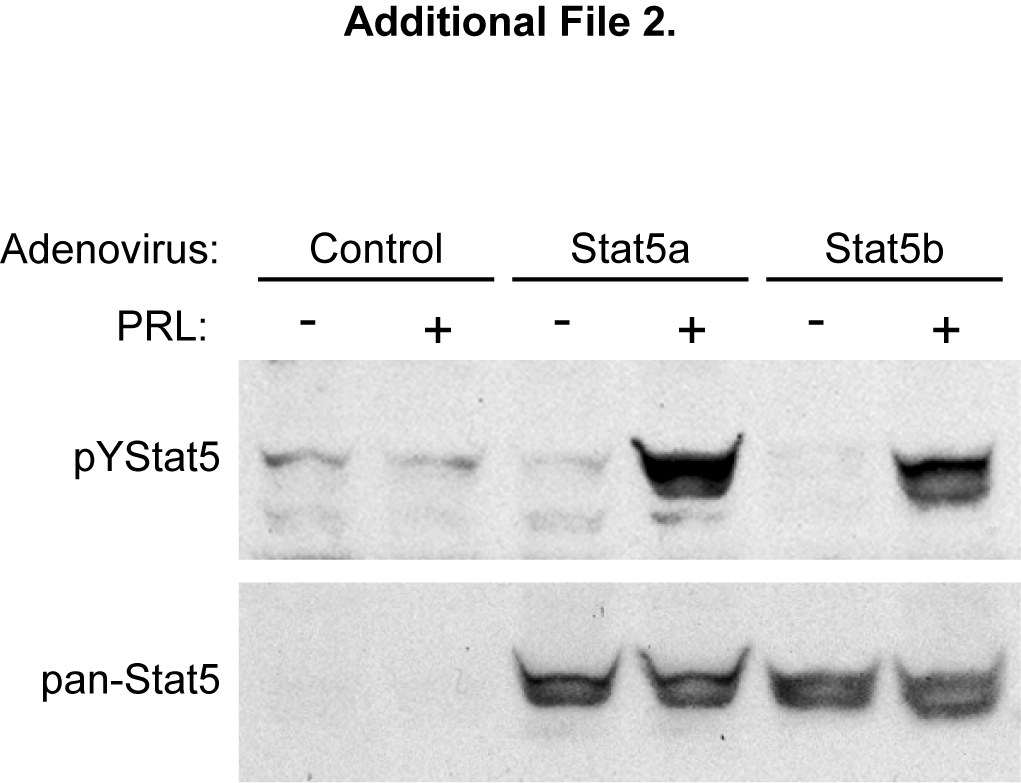

Supplement: Additional file 2 — Adenoviral expression and prolactin-induced phosphorylation of Stat5a and Stat5b in MCF7 human breast cancer cells lines. MCF7 cells were infected with adenovirus (MOI 40) expressing Stat5a or Stat5b and stimulated with prolactin for 20 minutes. Endogenous Stat5a and Stat5b were not detected with Western blot of whole-cell lysates from control cells, and phosphorylation of Stat5 was not detected in the absence of prolactin in control or Stat5a/Stat5b-overexpressing cells. [file bcr3328-S2.TIFF]
